# Patient perspectives of methadone formulation change in British Columbia, Canada: outcomes of a provincial survey

**DOI:** 10.1186/s13011-016-0048-3

**Published:** 2016-01-14

**Authors:** Alissa M. Greer, Sherry Hu, Ashraf Amlani, Sarah Moreheart, Olivia Sampson, Jane A. Buxton

**Affiliations:** University of British Columbia, School of Population and Public Health, 2206 East Mall, Vancouver, BC V6T 1Z3 Canada; BC Centre for Disease Control, 655 West 12th Avenue, Vancouver, BC V5Z 4R4 Canada; Simon Fraser University, 8888 University Drive, Burnaby, BC V5A 1S6 Canada

**Keywords:** Methadone, Methadose, Opioid substitution therapy, Medication formulation change, People who use drugs, Change intolerance, Policy change

## Abstract

**Background:**

In British Columbia, Canada, methadone maintenance treatment formulation transitioned from the oral liquid compound *Tang*™-flavoured methadone to the ten-times more concentrated cherry-flavoured Methadose™ in February 2014. We quantitatively describe perceptions and reported consequences among a sample of patients on methadone maintenance treatment following this transition.

**Methods:**

A province-wide survey was used. Bivariable analyses utilized independent samples *t*-tests, Phi associations, and Chi-square tests. Multivariable logistic regression analyses evaluated factors related to dependent variables – namely, increases in dose, pain, dope sickness, and the need to supplement with additional opioids.

**Results:**

Four hundred five methadone maintenance treatment patients from fifty harm reduction sites across British Columbia reported transitioning to Methadose™ in February 2014. The majority (*n* = 258; 73.1 %) heard about the formulation change from their methadone provider or pharmacist. Adjusted models show worse taste was positively associated with reporting an increasing dose (OR = 2.46; CI:1.31–4.61), feeling more dope sick (OR = 3.39; CI:1.88–6.12), and worsening pain (OR = 4.65; CI:2.45–8.80). Feeling more dope sick was positively associated with dose increase (OR = 2.24; CI:1.37–3.66), and supplementing with opioids (OR = 8.81; CI:5.16–15.05).

**Conclusions:**

Methadone maintenance treatment policy changes in British Columbia affect a structurally vulnerable population who may be less able to cope with transitions and loss of autonomy. There may be a psychosocial component contributing to the perception of Methadose™ tasting worse, and increased dope sickness, pain, and dose. Our study shows the pronounced negative impacts medication changes can have on patients without informed, coordinated efforts. We stress the need to engage all stakeholders allowing for communication about the reasons, risks and consequences of medication policy changes and provision of additional psychosocial support.

## Background

Methadone is a long-acting synthetic mu-opioid agonist with unique dual opioid receptor agonist and non-selective N-methyl-D-aspartate (NMDA) receptor antagonist abilities [1]. Methadone has been available for more than 50 years and is viewed as an efficacious and safe agent for the treatment of chronic pain and opioid addiction [1–3]. Taken orally once daily, methadone is used to initiate and maintain abstinence from illicit opioid use by relieving craving, suppressing opioid abstinence syndrome, and attenuating the euphoric effects of illicit opioid use [4, 5]. Methadone maintenance treatment (MMT) is an evidence-based harm reduction intervention shown to decrease injection drug use, and thereby reduces the impact of blood-borne illnesses, such as HIV and hepatitis C [6, 7], and reduces morbidity, and mortality among people who use drugs (PWUD) [8]. When patients with opioid dependence initiate MMT, methadone is initially administered on a daily basis under the supervision of a pharmacist. Doses are titrated by the prescribing physician, and split doses are considered in symptomatic patients who demonstrate rapid metabolism or who are pregnant [9]. Patients who demonstrate biopsychosocial stability may be granted “*carry*” privileges and receive doses of methadone for home administration [10].

Periodic transitions in drug dispensing and prescribing policies are necessary due to developments in pharmacological research or guideline shifts. In British Columbia (BC), Canada, several bodies regulate the dispensation of methadone and coordinate to implement policy or prescription changes: the BC Ministry of Health, a branch of the provincial government that leads policy changes regarding methadone; BC PharmaCare, a public entity covers the cost of prescription drugs, including methadone, for low-income individuals [11]; the College of Physicians and Surgeons of BC, which grants physicians the authority to prescribe methadone [12]; and the BC College of Pharmacists who standardize the training of pharmacists and dispensation of methadone in the province [13].

In 2014, these regulatory bodies made the decision to transition from the previous *Tang™*-flavoured (orange type flavor) 1 mg/mL pharmacist-compounded methadone formulation to methadone hydrochloride 10 mg/mL cherry-flavoured oral liquid (Methadose™) [10]. BC was the second province in Canada to implement the transition to Methadose™; 14,662 patients were registered in the methadone treatment program at the time [14]. The decision was based on several advantages of Methadose™, including a longer shelf life (up to four years if unopened), elimination of the need for refrigeration, improved quality control, and consistent dosing, which is possible mainly because Methadose™ is a solution prepared by the manufacturer rather than mixed or diluted by a pharmacist [10]. The new formulation is cited as hypertonic and painful to inject, a feature that may deter opioid abuse and medication diversion [10, 15]. In addition to formulation changes, a new policy was introduced by the College of Physicians and Surgeons of BC restricting pharmacies to provide home deliveries “under extraordinary circumstances,” requiring written authorization from the prescribing physician [10].

The transition to the methadone formulation Methadose™ posed several major public health concerns. First, methadone formulation changes may cause stress and instability in previously stable patients [16]. Such changes may result in patients discontinuing MMT and/or supplementing with other illegally obtained drugs [16, 17]. People who are prescribed MMT often belong to a vulnerable population that may experience difficulties with drug policy transitions and consequently, are less tolerant to changes in methadone prescribing or dispensing practices [16, 17]. Secondly, accidental overdoses during the transition period were a concern. Methadose™ is ten times more concentrated by volume compared to the previously compounded methadone, and is dispensed undiluted. Hence, there is potential for prescribing and dispensing errors during the transition period, further increasing overdose risk. As well, given that a smaller volume is dispensed, it may make it more difficult to titrate doses to patients.

During the methadone transition period in BC, pharmacists were asked to educate patients about safety concerns, including increased risk of overdose, education on take-home naloxone (an opioid antagonist), and appropriate security and storage [10]. The College of Physicians and Surgeons of BC in collaboration with the College of Pharmacist of BC provided information and training to physicians and pharmacists in the province; the colleges worked with people prescribed methadone to produce patient information resources [12]. Public health campaigns, such as posters, were distributed to harm reduction sites and media releases were initiated in BC to communicate the changes in appearance and concentration of the new methadone formulation [15].

PWUD who engaged with harm reduction sites and community advocacy groups expressed concerns regarding the lack of awareness and involvement in the transition process. Patients on MMT reported dissatisfaction with Methadose™ and disruptions in treatment, which informed the intent and need to conduct a quantitative province-wide survey. Our study seeks to understand experiences reported by MMT patients after the transition to the new methadone formulation, Methadose™. We compare the perceptions of the new Methadose™ to the previous compounded methadone formulation through several variables, including satisfaction and efficacy measures such as taste, pain, being dope sick, and dose changes.

## Methods

### Survey tool development

Four focus groups (*n* = 32) were undertaken with PWUD in Vancouver in the six weeks following the transition to Methadose™ to explore concerns about the methadone formulation change and inform the quantitative survey questions. The results of this qualitative study have been presented elsewhere [18]. Drug user organizations including the Vancouver Area Network of Drug Users and BC Association of People on Methadone were actively engaged and provided input regarding content and wording of the draft questions which were incorporated into the final survey. The final survey consisted of twelve methadone-specific questions, including: How do you find the taste of the new cherry Methadose™ compared to the orange methadone? Did your dose change with the new cherry Methadose™ compared to the orange methadone? Do you feel you need to take additional opioids with the new cherry Methadose™ compared to the orange methadone? In general, do you wake up feeling dope sick on the new cherry Methadose™ compared to the orange methadone? In general, how do you find the new cherry Methadose™ addresses your pain needs compared to the orange methadone?

The main variables of interest were subjective changes in dose, pain, taste, dope sickness, and the need to supplement with additional opioids. We also collected information on age, gender, ethnicity, housing status, geographic location, health authority, and how the participant heard about the Methadose™ changes (i.e. methadone health care provider or non-health care provider). Participants who reported carries were also asked how they stored Methadose™.

### Recruitment and sample

An annual survey was introduced in 2012 to obtain information about drug use and related harms, and to evaluate the harm reduction supply distribution program throughout BC [19]. A two-stage enrollment process was used: harm reduction distribution sites with sufficient clients and resources were identified and invited to participate. In 2014, sites were requested to recruit a maximum of 40 PWUD aged 19 years and older over eight weeks in the summer (i.e. four to six months after the Methadose™ transition occurred); Vancouver Coastal Health Authority was intentionally oversampled due to the high density population of MMT patients in this region. Sites were provided with $5 per survey to defray the costs of administering the survey and/or for participant incentives as the site determined most appropriate. Survey responses were entered into Fluid Surveys, an online database. Details of these survey methods have been described previously [19].

### Statistical analysis

Statistical analysis was performed with SPSS 21.0 and R 2.3.1. Bivariable evaluations of the data were conducted through independent samples *t* tests for continuous variables, and by Chi-square (*x*^*2*^) tests and calculating the Phi coefficient of association to determine the relationship between binary variables. Multivariable logistic regression analyses were performed to evaluate factors related to dependent variables – namely: dose, pain, dope sickness, and the need to supplement with additional opioids. First, independent variables were considered in a forward stepwise variable selection procedure. The criterion for entry in the stepwise procedure was a significance level of *P* less than 0.05. Two explanatory variables, pain and dope sickness, were not included as predictors in any same model. The rationale for this was threefold. First, the bivariable analysis revealed a relatively strong association (*Phi* = 0.46), indicating that they may be describing the same effects, as pain may be a symptom of dope sickness. Second, the addition of pain increased the standard error of the beta coefficient for dope sick, indicative of a collinear variable. Finally, adding pain into the models in addition to dope sickness decreased the overall predictive power of the model, as indicated in the Hosmer Lemeshow tests. Dope sickness was retained in the final model of supplement and dose increase since the predictive power was higher than the model that included pain instead. The Hosmer Lemeshow chi-square tests (df = 8) for all models suggested they were a good fit to the data as *p* = 0.29–0.89 (>0.05). Nagelkerke R^2^ for models ranged from 0.10 to 0.60 indicating models were able to explain between 10–60 % of variability in the data. Although age and gender did not have a significant effect on any dependent variable, both were force entered into the models to account for any influence of demographics. For responses where participants could qualitatively enter an “other” description, items were categorized and frequencies were calculated to describe patterns.

## Results

Fifty harm reduction distribution sites across the province returned 1322 completed surveys. Of these, 405 respondents reported being on MMT at the time of transition to Methadose™. The survey provided a sample of MMT patients from all five geographic health regions across BC. The proportion of respondents from each regional health authority that were enrolled in an MMT program varied from 39.5 % in Vancouver Coastal Health to 19.0 % in Fraser Health Authority. Eighty-nine percent of participants in this sample reported polysubstance use in the past 7 days. The socio-demographic characteristics of the 405 respondents in the MMT program at the time of transition are shown in Table 1.

**Table 1:** Sociodemographics of a sample of methadone maintenance treatment patients following a methadone formulation change in British Columbia (*n* = 405)

	Number	Percent
Region (*n* = 405)		
Fraser	40	9.9
Interior	46	11.4
Northern	20	4.9
Vancouver Coastal	232	57.3
Vancouver Island	67	16.5
Ethnicity (*n* = 393)		
Aboriginal	114	29.0
Non-Aboriginal	279	71.0
Gender (*n* = 401)		
Female	161	40.2
Male	237	59.1
Transsexual	3	0.7
Housing (*n* = 390)		
Stable (housed > =3 months)	305	78.2
Unstable (<3 months or homeless)	85	21.8
Age in years (*n* = 403)	Mean (SD)	Range
	42 (10.3)	19 – 71

The majority (80.9 %) of participants reported a worse taste with the new Methadose™ *cherry*-flavoured syrup compared to the previous *Tang*™-flavoured methadone (Table 2). A third of respondents reported increasing their dose of Methadose™, while over half of respondents reported having worse pain, feeling more dope sick, and supplementing their methadone with other opioids. Table 3 describes variables assessing MMT outcomes. The majority (*n* = 258; 73.1 %) of participants heard about the changes in their MMT from their health care provider or methadone pharmacist. Thirty-two (16.1 %) patients with methadone carries (*n* = 199) stored their methadone in a locked box. Of those who provided a reason for increasing their methadone dose (*n* = 76), three quarters reported it was to manage symptoms; 46.1 % to avoid feeling dope sick and 28.9 % to relieve pain.

**Table 2:** Perceptions and outcomes of patients on methadone maintenance treatment following a formulation change

	N (%)
How heard about the change (*n* = 353)	
Health care provider or methadone pharmacist	258 (73.1)
Non methadone service provider	33 (9.3)
Family or friend	26 (7.4)
Newspaper or poster	36 (10.2)
Of those with carries (*n* = 199)	
Does not store methadone in locked box	167 (83.9)
Does store methadone in locked box	32 (16.1)
Splitting doses (*n* = 293)	
No change	269 (91.8)
Started splitting	21 (7.2)
Stopped splitting	3 (1.0)
Reason dose increased (*n* = 76)	
To avoid feeling dope sick	35 (46.1)
To relieve pain	22 (28.9)
To avoid using other opioids	19 (25.0)
Reason for supplementing (*n* = 178)	
To avoid feeling dope sick	92 (51.7)
To relieve pain	86 (48.3)
Taste difference (*n* = 371)	
Same or better taste	71 (19.1)
Worse taste	300 (80.9)
Dose changes (*n* = 320)	
Lowered or same dose	213 (66.6)
Increased dose	107 (33.4)
Dope sick changes (*n* = 358)	
Same or less dope sick	156 (43.6)
More dope sick	202 (56.4)
Pain changes (*n* = 358)	
Same or better pain	165 (46.1)
Worse pain	193 (53.9)
Supplements with opioids (*n* = 360)	
No supplementing with opioids	179 (49.7)
Yes supplementing with opioids	181 (50.3)

**Table 3:** Bivariable associations between taste, dose increased, supplements, pain, and dope sickness following a methadone formulation change

	Taste worsened		Dose increased		Began supplementing with opioids		More dope sick	
	X^2^	Phi	X^2^	Phi	X^2^	Phi	X^2^	Phi
Dose increased	9.93**	0.20**						
Began supplementing with opioids	3.99*	0.11*	21.50**	0.27**				
More dope sick	22.52**	0.25**	13.70**	0.20**	70.63**	0.46**		
More pain	22.23**	0.27**	13.06**	0.21**	29.43**	0.30**	77.28**	0.46**

*p < 0.05, **p < 0.01

degrees of freedom for Chi-square (X^2^) tests = 1

In the bivariable analyses, outcome and explanatory variables of interest were all significantly and strongly associated as indicated in the Chi-square tests and *Phi* association measure (Table 3). In the multivariable logistic regression analysis (Table 4), reporting worse taste was independently associated with increased dose (OR = 2.46; CI: 1.31–4.61), more dope sick (OR = 3.39; CI: 1.88–6.12) and worse pain (OR = 4.65; CI: 2.45–8.80), while being more dope sick was found to be positively associated with the odds of increasing dose (OR = 2.24; CI: 1.37–3.66), and supplementing with opioids (OR = 8.81; 5.16–15.05).

**Table 4:** Adjusted odds ratios (OR) with 95 % confidence intervals (CI) and goodness of fit for taste and more dope sick related to odds of dose increase, supplements with additional opioids, more dope sick, and more pain following methadone formulation change

	Wald	OR*	95 % CI**	Hosmer Lemeshow Test	Nagelkerke R^2^
Dose Increased					
Taste	7.79	2.46	1.31–4.61	0.89	0.37
More dope sick	10.28	2.24	1.37–3.66		
Supplements					
More dope sick	63.89	8.81	5.16–15.05	0.29	0.60
More dope sick					
Taste	11.91	3.39	1.88–6.12	0.30	0.11
More pain					
Taste	22.06	4.65	2.45–8.80	0.83	0.10

^*^OR adjusted for age, gender, and indicated covariates

**95 % CI based on Wald chi-square tests with df = 1

## Discussion

The goal of this study was to explore the perceptions of how persons enrolled in MMT in BC were affected by the transition to Methadose™. Our findings revealed large proportions of participants reported worse taste, the need to increase methadone dose, and increased withdrawal and pain symptoms. Worse taste was significantly correlated with reported lack of effectiveness and deterioration of symptoms across multiple parameters, as demonstrated by more withdrawal symptoms, pain and need to increase dose. These associations were not significantly related to how patients heard about the formulation change, geographic region, or other sociodemographic factors. Overall, our findings of increased pain, withdrawal symptoms, and need to increase methadone dose following the formulation change to Methadose™ have implications for the wellbeing of MMT patients in BC that must be considered.

Previous studies support our findings that medication transition periods can have pronounced bio-psychosocial impacts on patients. In the United Kingdom, change in methadone formulation in 1992 was correlated with an increase use of non-prescription opioids, decline in social stability, and an increase in pharmacy break-ins [20]. A clinical study conducted by Soyka and Zingg in 2009 found the transfer from racemic methadone to (R)-methadone decreased withdrawal symptoms, cravings, and supplementing with additional drugs [21]. In our study, over half of participants reported supplementing with other drugs following the transition to Methadose™. Methadone diversion and its illicit use are not uncommon; the 2014 survey of clients at harm reduction supply distribution sites in BC found that 8 % of respondents accessed methadone without a prescription (unpublished BCCDC data). Diversion in medication compliance has been critical in subsequent injection drug use, crime, and morbidity and mortality rates [22, 23]; hence, any instability in MMT leading to a rebound in illicit drug use may result in possible decline in social function and worse health outcomes in BC.

Silver and Shaffer (1997) found patients could be more intolerant to methadone formulation changes than others (coined ‘change intolerance’) [24]. In their study of 177 MMT patients, researchers found gender, treatment history, and previous methadone abuse predicted change intolerance. Gourevitch et al. provides further evidence demonstrating that change intolerance to different methadone formulations is related to psychosocial stressors rather than having a pharmacodynamic basis [17]. In a small double-blind crossover trial of three methadone formulations they demonstrated self-reported withdrawal symptoms did not correlate with plasma methadone levels [17]. The effect of taste on patients’ perception of pharmacological efficacy and side effects is not well studied. A randomized control trial conducted by Farr (1986) showed that medication taste affected participants’ perceptions [25]. In this study, bitter tasting zinc gluconate lozenges were perceived to have more negative side effects than the tasteless placebo tablets. This effect diminished when taste-matching placebos were used [25]. In France, where the capsule formulation of MMT recently became available in addition to the syrup formulation, two recent studies showed the potential superiority of the dry (capsule) MMT formulation [18, 26]. Boucherie et al. (2015) demonstrated that there was a rise in capsule MMT users in a five year period with a corresponding drop in prevalence of individuals using the syrup formulation [26], and Eiden et al. (2013) described 80 % of patients experienced negative side effects from use of the syrup MMT formulation [18]. In light of the large proportion of MMT patients in our study who reported worse taste and its relation to negative outcomes, consideration of other formulations of methadone including the capsule form, may be warranted. However, some literature has highlighted that non-syrup forms of MMT may increase diversion and misuse liability [18].

These findings are corroborated by a recent qualitative study conducted by McNeil et al. (2015) who interviewed 34 MMT patients in the Vancouver region; 31 who had transitioned to Methadose^TM^ at the time of interview [16]. This study supports the notion that the methadone formulation transition in BC produced considerable negative health and social impacts on patients and demonstrated that these effects were exacerbated by the structural vulnerability that patients on MMT are subjected to [16]. They also suggest real or perceived withdrawal symptoms might have a psychological component, as in our study, which strongly suggests perceptions of changes in taste contribute to withdrawal symptoms, pain, and need to increase dose. Although we anticipated similar findings, our study had a number of strengths and adds to the literature. We had quantitative responses from over 400 people throughout the province and, thus, our results were not limited to the Vancouver area. While methadone formulation changes have occurred in other provinces in Canada (i.e. Alberta, Ontario), there remains a lack of evaluation data of the impact and differences of the implementation of these policies.

These studies and ours underscore the importance of recognizing that many persons participating in MMT belong to a particularly vulnerable population who are often victim to structural discrimination, which may make them less able to cope with imposed medication changes and loss of autonomy [25]. Trujols et al. (2011) argue much of the satisfaction with MMT comes from patients who perceive themselves as participating to some extent in treatment decisions [27]. The impact of the transition to Methadose™ in BC among MMT patients may be further exacerbated by the change in home delivery policy further adding to the instability and dissatisfaction to the program. The capacity for agency and a flexible interim period appear to be important factors in the stability of medication transitions. There is evidence that patients are more satisfied with their MMT programs when they perceive themselves as participating in treatment decision-making [27]. Another study suggested that although there may be some transitional instabilities encountered after methadone formulation changes, they could be further mitigated by providing patients with a longer transition period as well as a choice of the transitional process [28].

One strategy to mitigate this impact that could be incorporated during medication formulation changes, is involving and consulting the community affected in the development and implementation of new regulatory policies. El Ansari and Phillips (2004) found that a low amount of involvement with the community is associated with decreased costs and increased satisfaction with policies [29]. In BC, the College of Pharmacists, College of Physicians and Surgeons and Ministry of Health met with representatives from the Vancouver Area Network of Drug Users and BC Association of People on Methadone on three occasions over ten months prior to the change to Methadose™ (Personal communication Solven S., 2015). During this time patient groups provided feedback regarding the dispensing standards for MMT that resulted in several changes and helped to produce communication materials (i.e. “Think before you drink” campaign) [30].

Despite a concerted effort by the College of Pharmacists of BC to engage with people on methadone, our study found significant negative perceptions of the methadone formulation change in the province. It is interesting to note that our results showed nearly three-quarters heard about the methadone formulation transition from their methadone-prescribing physician or pharmacist, who could be a useful avenue for the dissemination of educational resources. However, the remaining one-quarter of participants could have benefited from greater awareness and public education campaigns during the transition period. Patients may benefit from longer and more flexible transitional timeline of several months, rather than weeks, when the old formulation may be accessible during the introduction of the new formulation. During this time, patients could be monitored closely for changes in pain and dope sickness. Although patients may be more accepting with a longer and more flexible transition period, the College of Pharmacists of BC believed a shorter transition period would minimize the chance of error with the two different strengths, and therefore decided against an increased transition period(Personal communication Solven S., 2015). Lastly, it may have been helpful to introduce different formulation options for patients; similar to Alberta and Ontario, Methadose™ may be better tolerated (particularly taste) if MMT patients were given the option to have unflavored Methadose™ or be able to dilute in a *Tang*™-flavored drink which would maintain consistency with previous volume and flavor [31, 32].

While our study highlights some important findings that may have policy implications, several limitations must be considered. First, we recruited and sampled people who access harm reduction sites for supplies across BC, signifying that many of our survey participants may use illicit drugs in addition to their MMT. These patients may represent a structurally vulnerable population [16]; as such, these findings may not be generalizable to all MMT patients. However, it is important to understand the challenges of MMT for these individuals and reduce their heightened barriers for access [33]. Although our study was administered in all five regional health authorities at 50 harm reduction sites across BC, access to MMT varies across regions. Regions where patients have one source of MMT may be more accepting of changes as they are not accustomed to having a choice of MMT; however, our findings were not significantly different across regions. As this study was a cross-sectional design after the change occurred, satisfaction with MMT was not assessed prior to the change. Future research may benefit from measuring dose and satisfaction with MMT longitudinally, capturing both before and after measures. This study did not examine whether negative experiences following the formulation change impacted continuation on MMT, which could be an area for future research. Finally, multiple bivariate analyses were conducted with many variables serving as both explanatory and outcome variables. With multiple analyses, we acknowledge the consequent risk of Type I error that may be inflated with this type of analysis.

## Conclusion

In conclusion, we characterized several negative perceptions and outcomes among a sample of MMT patients following a province-wide methadone formulation change to Methadose™ in BC, Canada. These findings highlight the structural vulnerability PWUD and MMT patients face, and point to the importance of involving all stakeholders policy decision-making. We urge policy makers to consider a more engaging and transparent approach when working with structurally vulnerable populations, including people with a history of substance misuse, incarceration and mental health illness, and homelessness.

## Ethics, consent and permissions

This study was approved by the University of British Columbia Research Ethics Board prior to collecting data, and for the duration of this research. Informed consent was obtained from all participants, including consent to publish.

## Abbreviations

BC: British Columbia
CI: Confidence interval
MMT: Methadone maintenance treatment
OR: Odds ratio
PWUD: People who use drugs

## References

[1] Layson-Wolf C, Goode J-V, Small RE (2002). Clinical use of methadone. J Pain Palliat Care Pharmacother..

[2] Fugelstad a, Stenbacka M, Leifman a, Nylander M, Thiblin I (2007). Methadone maintenance treatment: The balance between life-saving treatment and fatal poisonings. Addiction.

[3] Mattick RP, Breen C, Kimber J, Davoli M (2009). Methadone maintenance therapy versus no opioid replacement therapy for opioid dependence. Cochrane Database Syst Rev Online..

[4] Marshall Z, Dechman MK, Minichiello A, Alcock L, Harris GE (2015). Peering into the literature: A systematic review of the roles of people who inject drugs in harm reduction initiatives. Drug Alcohol Depend..

[5] Marsch L a (1998). The efficacy of methadone maintenance interventions in reducing illicit opiate use, HIV risk behavior and criminality: a meta-analysis. Addict Abingdon Engl.

[6] Reddon H, Milloy MJ (2014). Simo a., Montaner J, Wood E, Kerr T. Methadone maintenance therapy decreases the rate of antiretroviral therapy discontinuation among HIV-positive illicit drug users. AIDS Behav.

[7] Harris M, Rhodes T (2013). Methadone diversion as a protective strategy: The harm reduction potential of “generous constraints.”. Int J Drug Policy..

[8] Buxton J (2010). a., Kuo ME, Ramji S, Yu A, Krajden M. Methadone use in relation to hepatitis C virus testing in British Columbia. Can J Public Health..

[9] Methadone Maintenance Program: Clinical Practice Guidelines. College of Physicians and Surgeons. 2014. https://www.cpsbc.ca/files/pdf/MMP-Clinical-Practice-Guideline.pdf Accessed 28 Sep 2015.

[10] Policy Guide (2013). Methadone Maintenance Therapy.

[11] PharmaCare for B.C. Residents. Goverment of British Columbia. 2015. http://www2.gov.bc.ca/gov/content/health/health-drug-coverage/pharmacare-for-bc-residents. Accessed 28 Sep 2015.

[12] Methadone Maintenance Program: Clinical Practice Guidelines. Coll Phys Surg BC; 2014. https://www.cpsbc.ca/files/pdf/MMP-Clinical-Practice-Guideline.pdf. Accessed 28 Sep 2015.

[13] Professional Practice Policy - 71: Delivery of Methadone for Maintenance. College of Pharmacists of British Columbia; 2013. http://library.bcpharmacists.org/A-About_Us/A-2_Governance/5003-PGP-PPP71.pdf Accessed 28 Sep 2015.

[14] Annual Report 2013–2014. Coll Phys Surg BC. 2013. https://www.cpsbc.ca/files/pdf/2013-14-Annual-Report.pdf Accessed 28 Sep 2015.

[15] British Columbia Harm Reduction Program. BCCDC. http://towardtheheart.com. Accessed 28 Sep 2015.

[16] McNeil R, Kerr T, Anderson S, Maher L, Keewatin C, Milloy MJ (2015). Negotiating structural vulnerability following regulatory changes to a provincial methadone program in vancouver, canada: A qualitative study. Soc Sci Med..

[17] Gourevitch MN, Hartel D, Tenore P, Freeman K, Marion I, Hecht J (1999). Three oral formulations of methadone: A clinical and pharmacodynamic comparison. J Subst Abuse Treat..

[18] Eiden C, Bertomeu L, Clavel V, Faillie JL, Petit P, Peyriere H (2013). New formulation of methadone for opioid dependence in France: acceptability and diversion/misuse liability. Therapie.

[19] Kuo M, Shamsian A, Tzemis D, Buxton JA (2014). A drug use survey among clients of harm reduction sites across British Columbia, Canada, 2012. Harm Reduct J..

[20] Steels MD, Hamilton M, McLean PC (1992). The consequences of a change in formulation of methadone prescribed in a drug clinic. Br J Addict..

[21] Soyka M, Zingg C (2009). Feasability and safety of transfer from racemic methadone to (R)-methadone in primary care: clinical results from an open study. World J Biol Psychiatry..

[22] Kelly SM, O’Grady KE, Mitchell SG, Brown BS, Schwartz RP (2011). Predictors of methadone treatment retention from a multi-site study: a survival analysis. Drug Alcohol Depend..

[23] Ball JC, Ross A (1992). The Effectiveness of Methadone Maintenance Treatment. N Engl J Med..

[24] Silver JS, Shaffer HJ (1997). Change intolerance to shifts in methadone formulation: A preliminary investigation. J Subst Abuse Treat..

[25] Farr BM, Gwaltney JM (1987). The problems of taste in placebo matching: an evaluation of zinc gluconate for the common cold. J Chronic Dis..

[26] Boucherie Q (2015). New methadone formulation in France: results from 5 years of utilization. Therapie..

[27] Trujols J (2012). Patient satisfaction with methadone maintenance treatment: the relevance of participation in treatment and social functioning. Drug Alcohol Depend..

[28] Myton T (2003). Descriptive study of the effects of altering formulation of prescribed methadone from injectable to oral. Psychiatr Bull..

[29] El Ansari W, Phillips C (2004). The costs and benefits to participants in community partnerships: a paradox? Health Promot Pract. Jan.

[30] Think before you drink. BC College of Pharmacists; 2014. http://library.bcpharmacists.org/A-About_Us/A-8_Key_Initiatives/5138-MMT_Patient_Poster_8_5x11.pdf. Accessed 28 Sep 2015.

[31] Alberta College of Pharmacists. ODT Guidelines: Medication-Assisted Treatment for Opioid Depednency Guidelines for Pharmacists and Pharmacy Technicians. https://pharmacists.ab.ca/system/files/ODTGuidelines.pdf. Accessed 28 Sep 2015.

[32] Methadose and the Policy for Methadone Maintenance Treatment Reimbursement under the Ontario Drug Benefit Program. 2014. http://www.health.gov.on.ca/en/pro/programs/drugs/opdp_eo/notices/cmmt_faq_pharmacists_20140620.pdf. Accessed 28 Sep 2015.

[33] Strike CJ, Gnam W, Urbanoski K, Fischer B, Marsh DC, Millson M (2005). Factors predicting 2-year retention in methadone maintenance treatment for opioid dependence. Addict Behav..

